# Impact of spaceflight on the murine thymus and mitigation by exposure to artificial gravity during spaceflight

**DOI:** 10.1038/s41598-019-56432-9

**Published:** 2019-12-27

**Authors:** Kenta Horie, Tamotsu Kato, Takashi Kudo, Hiroki Sasanuma, Maki Miyauchi, Nobuko Akiyama, Takahisa Miyao, Takao Seki, Tatsuya Ishikawa, Yuki Takakura, Masaki Shirakawa, Dai Shiba, Michito Hamada, Hyojung Jeon, Nobuaki Yoshida, Jun-ichiro Inoue, Masafumi Muratani, Satoru Takahashi, Hiroshi Ohno, Taishin Akiyama

**Affiliations:** 1 Laboratory for Immune Homeostasis, RIKEN Center for Integrative Medical Sciences, Yokohama, 230-0045 Japan; 2 Laboratory for Intestinal Ecosystem, RIKEN Center for Integrative Medical Sciences, Yokohama, 230-0045 Japan; 3 Laboratory Animal Resource Center in Transborder Medical Research Center, and Department of Anatomy and Embryology, Faculty of Medicine, University of Tsukuba, Ibaraki, 305-8575 Japan; 4 Mouse Epigenetics Project, ISS/Kibo experiment, Japan Aerospace Exploration Agency (JAXA), Ibaraki, 305-8505 Japan; 5 Laboratory of Developmental Genetics, Institute of Medical Science, University of Tokyo, Tokyo, 108-8639 Japan; 6 Laboratory for Immunogenetics, RIKEN Center for Integrative Medical Sciences, Yokohama, 230-0045 Japan; 7 JEM Utilization Center, Human Spaceflight Technology Directorate, JAXA, Ibaraki, 305-8505 Japan; 8 Division of Cellular and Molecular Biology, Institute of Medical Science, University of Tokyo, Tokyo, 108-8639 Japan; 9 Transborder Medical Research Center, and Department of Genome Biology, Faculty of Medicine, University of Tsukuba, Ibaraki, 305-8575 Japan

**Keywords:** Thymus, Physiology, Computational biology and bioinformatics

## Abstract

The environment experienced during spaceflight may impact the immune system and the thymus appears to undergo atrophy during spaceflight. However, molecular aspects of this thymic atrophy remain to be elucidated. In this study, we analysed the thymi of mice on board the international space station (ISS) for approximately 1 month. Thymic size was significantly reduced after spaceflight. Notably, exposure of mice to 1 × *g* using centrifugation cages in the ISS significantly mitigated the reduction in thymic size. Although spaceflight caused thymic atrophy, the global thymic structure was not largely changed. However, RNA sequencing analysis of the thymus showed significantly reduced expression of cell cycle-regulating genes in two independent spaceflight samples. These reductions were partially countered by 1 × *g* exposure during the space flights. Thus, our data suggest that spaceflight leads to reduced proliferation of thymic cells, thereby reducing the size of the thymus, and exposure to 1 × *g* might alleviate the impairment of thymus homeostasis induced by spaceflight.

## Introduction

Astronauts experience several environmental changes during space flight such as hypergravity during launching, microgravity, psychological stress, and high-dose space radiation. These hostile environmental changes affect various physiological functions of astronauts^1–4^. Regarding the immune system, various latent viruses were reportedly re-activated^5,6^. Analysis of blood samples from astronauts suggested that spaceflight influences the distribution of leukocytes^7,8^, and natural killer cell^9^, granulocyte and monocyte function^7,10^, along with plasma cytokine levels^11,12^. Consequently, these studies suggested that spaceflight impacts on the immune system. However, the effect of spaceflight on lymphoid organs and tissues are generally difficult to evaluate in humans. Therefore, animal models, mainly rodents, have been used to address this issue in spaceflight experiments^13,14^.

The thymus is a primary lymphoid organ generating almost all T cells in the body. Disruption of thymic function causes immune deficiency and autoimmune diseases. In addition to age-dependent atrophy, the thymus undergoes atrophy by various psychological stressors, radiation, and virus infection^15–17^. Normally, double positive CD4^+^CD8^+^ thymocytes (DPs) are the most sensitive to these insults^16,17^. A short-term hindlimb unloading experiment, which is a ground model of spaceflight^13,18^, causes apoptosis of DPs in the cortex region of the thymus^19,20^. Indeed, several studies using rodents revealed thymic atrophy after spaceflight^21–23^. Consistent with this finding, in humans, T cell receptor excision circle (TREC) PCR assay showed that T cells newly generated from the thymus were decreased in the blood of astronauts by spaceflight^24^, supporting the impact of spaceflight on thymus homeostasis.

Artificial gravity was proposed to be used as a countermeasure for alleviating weightlessness-induced problems during spaceflight^25^. However, to date, evaluation of its effect is difficult in humans during spaceflight. The Japan Aerospace Exploration Agency (JAXA) developed an experimental platform, the Multiple Artificial-gravity Research System (MARS), in which mouse cages can be centrifuged to control the gravity experienced in the International Space Station (ISS)^26,27^. JAXA executed several missions to perform mouse experiments using MARS on board the ISS^14,26,27^. In these missions, groups of mice were exposed to 1 × *g* during spaceflight to help clarify the effect of artificial 1 × *g* exposure on adverse events induced by spaceflight.

The aim of this study is to elucidate the impact of spaceflight on the thymus at the molecular level and to test the effect of artificial 1 × *g* exposure on spaceflight-induced changes in the thymus. We analysed the thymi of mice recovered from the JAXA missions using MARS (missions MHU-1 and MHU-2)^26,27^. Deep sequencing of thymus cDNA suggested that the thymic atrophy caused by spaceflight might be ascribed to reduced proliferation of thymic cells. Moreover, the spaceflight-induced atrophy and changes in gene expression in the thymus were significantly rescued by exposure to 1 × *g* during spaceflight.

## Results

### Spaceflight induces thymic involution, which is partially rescued by 1 × *g* exposure

To elucidate the impact of spaceflight on thymus homeostasis and the effect of 1 × *g* exposure on spaceflight-induced changes in the thymus, we analysed the thymi of mice housed in MARS on board the ISS. In the first mission (MHU-1)^14,26^, 12 mice were housed in the ISS for 35 days. Six of these mice were exposed to artificial 1 × *g* (AG group) by centrifuging the mouse cage in the ISS, with the remaining six being exposed to microgravity throughout the duration of the flight (MG group). Thymi were collected 2 days after landing on earth. A further six mice on the same genetic background were housed on earth in identical cages to those used in MHU-1 as ground control (GC).

**Figure 1:**
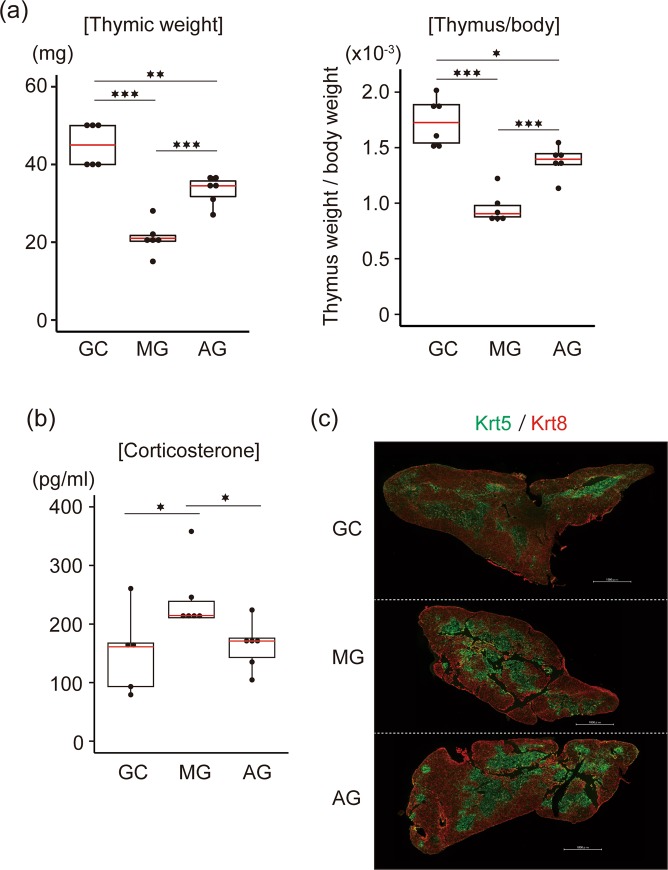
Influence of spaceflight and 1 × *g* exposure during spaceflight on thymus weight, plasma corticosterone, and global thymic structure. (**a**) Thymus weight and ratio of thymic weight to body weight in spaceflight and control mice. N = 6. Box and dot plots are shown. Red line indicates medians. GC, ground control mice; MG, mice flown in ISS; AG, mice receiving 1 × *g* in ISS. (**b**) Plasma corticosterone concentration. N = 6. Box and dot plots are shown. Red line indicates medians. Corticosterone concentration in plasma from spaceflight and control mice was determined by ELISA. (**c**) Immunohistochemical staining of thymic sections with Krt5 (green) and Krt8 (red). Scalebars indicate 1000 µm.

In addition to the reduction in body weight as previously reported^26^, thymic weight and thymus weight relative to body weight was reduced by approximately half in mice without centrifugation on board the ISS for 35 days (MG) compared with GC mice (Fig. 1a). Importantly, the relative thymus weight of AG mice was significantly higher than that of MG mice, indicating that exposure to 1 × *g* during spaceflight alleviates thymic atrophy. Interestingly, the relative thymus size of AG mice remained significantly smaller than GC mice (approximately 80% of thymus size in GC mice). This suggests that, besides gravity, other environmental factors during spaceflight might influence thymic size.

Many physiological and psychological stressors produce glucocorticoids through activation of the hypothalamic–pituitary–adrenal axis^28,29^. Up-regulation of corticosterone in the blood causes apoptosis of DPs in the cortex region of the thymus^30^. We therefore checked the corticosterone concentrations in the plasma of MG, AG and GC mice (Fig. 1b). Corticosterone concentration was increased approximately two-fold in MG compared with GC mice, and this was countered under artificial gravity conditions. Thus, this small increase may be due to stress or dehydration caused by microgravity.

Apoptosis of DP thymocytes frequently causes a reduction in the area of the thymic cortex region^30^. We analysed thymic sections from these mice by immunostaining (Fig. 1c). Immunostaining with anti-keratin-5 (Krt5; a marker for the medullar region) and keratin-8 (Krt-8; a marker of the cortex region) did not show a prominent change in the global structure and size of the medulla and cortex regions. Thus, a severe apoptosis of DP thymocytes was unlikely to have occurred in the thymi of mice experiencing spaceflight.

### Spaceflight causes a significant change in gene expression in the thymus

**Figure 2:**
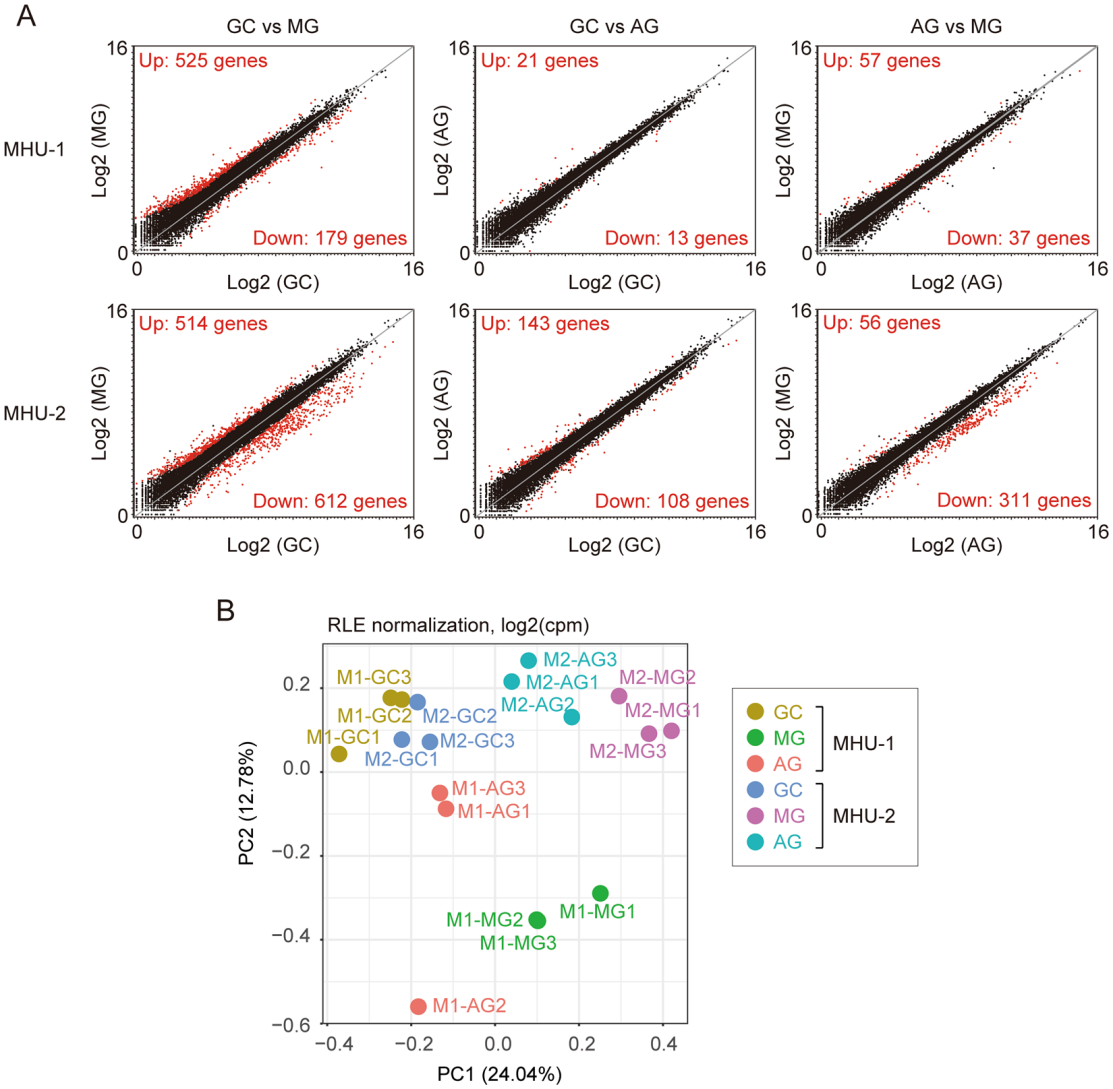
RNA-seq analysis of the thymus of spaceflown and ground control mice. (**A**) Scatter plots of RNA-seq data in MHU-1 and MHU-2 (N = 3 for each condition and mission). Each axis shows normalised log2 expression values. GC, ground control mice; MG, mice flown in ISS; AC, mice receiving 1 × *g* by centrifugation in ISS. Significantly up- or down-regulated genes are indicated as red dots. (**B**) PCA plots of RNA-seq data in both flights.

To address the molecular mechanisms underlying the change in thymic size induced by spaceflight, we performed RNA-seq analysis of thymi of MG, AG and GC mice. Two independent flight samples from JAXA missions using MARS (MHU-1 and MHU-2) were analysed to test reproducibility. Differences in flight parameters and schedules, transportation between MHU-1 and MHU-2 are summarised in Supplementary Table I. We compared gene expression profiles between MG, AG and GC mice in each mission (Fig. 2A,B). Data analysis suggested that expression levels of many genes were significantly changed in MG mice compared with GC counterparts. Moreover, gene profiles between AG and MG animals were considerably different. As expected, the difference in gene expression profile between GC and AG mice was much less than MG vs GC, and MG vs AG mice. Biological triplicate samples of each condition showed relatively similar gene expression profiles, except for one outlier in the AG group from MHU-1 (Fig. 2B).

Principal component analysis (PCA) suggested that GC samples were similar between the two missions (Fig. 2B). In contrast, there were considerable differences in gene expression profiles of AG and MG between the two missions (Fig. 2B), suggesting that differences in flight conditions and schedules (Supplementary Table 1) substantially influence the gene expression profile in the thymus.

### Spaceflight reproducibly induced reduction of cell cycle-related genes in the thymus

**Figure 3:**
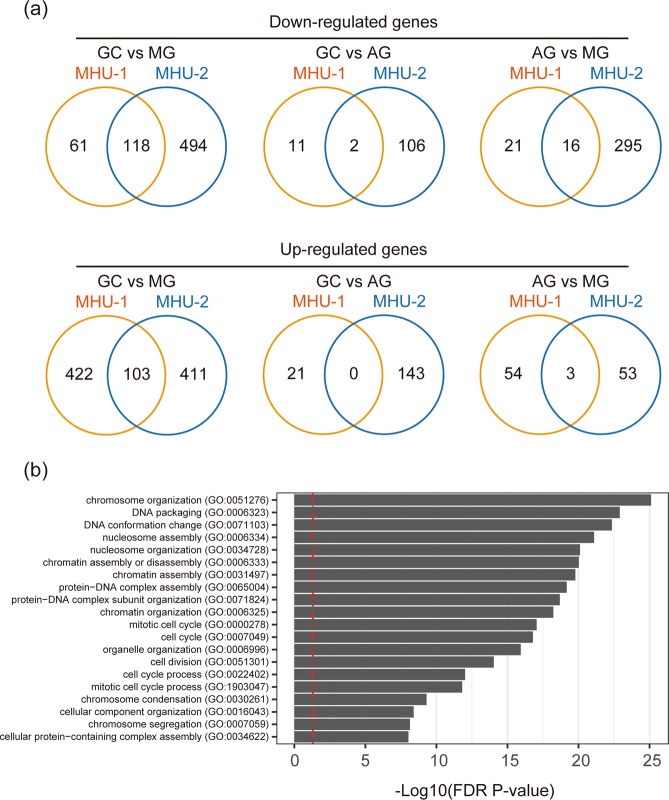
Numbers and GO analysis of shared changed genes between the two spaceflight experiments. (**a**) Venn diagrams of down-regulated (upper) and up-regulated (lower) genes in GC vs MG (left), GC vs AG (middle), and AG vs MG (right) animals in MHU-1 and MHU-2. (**b**) GO enrichment analysis of genes down-regulated in the thymus of MG mice compared with GC mice. The most significantly enriched 20 terms (low top 20 in FDR P-value) are shown. Red line indicates that FDR P = 0.05.

To determine events reproducibly occurring in the thymus during spaceflight, we identified genes changed in both MHU-1 and MHU-2 missions (Fig. 3a). A total of 118 genes were significantly down-regulated (false discovery rate [FDR] P < 0.05, two-fold change) in MG mice of MHU-1 and MHU-2 compared with respective GC. Moreover, 103 genes were up-regulated in MG animals of the two missions. Gene ontology (GO) analysis showed that genes related to the cell cycle and chromosome organisation were enriched in the down-regulated genes (Fig. 3b). We did not find significantly enriched terms in the set of genes up-regulated in MG mice.

**Figure 4:**
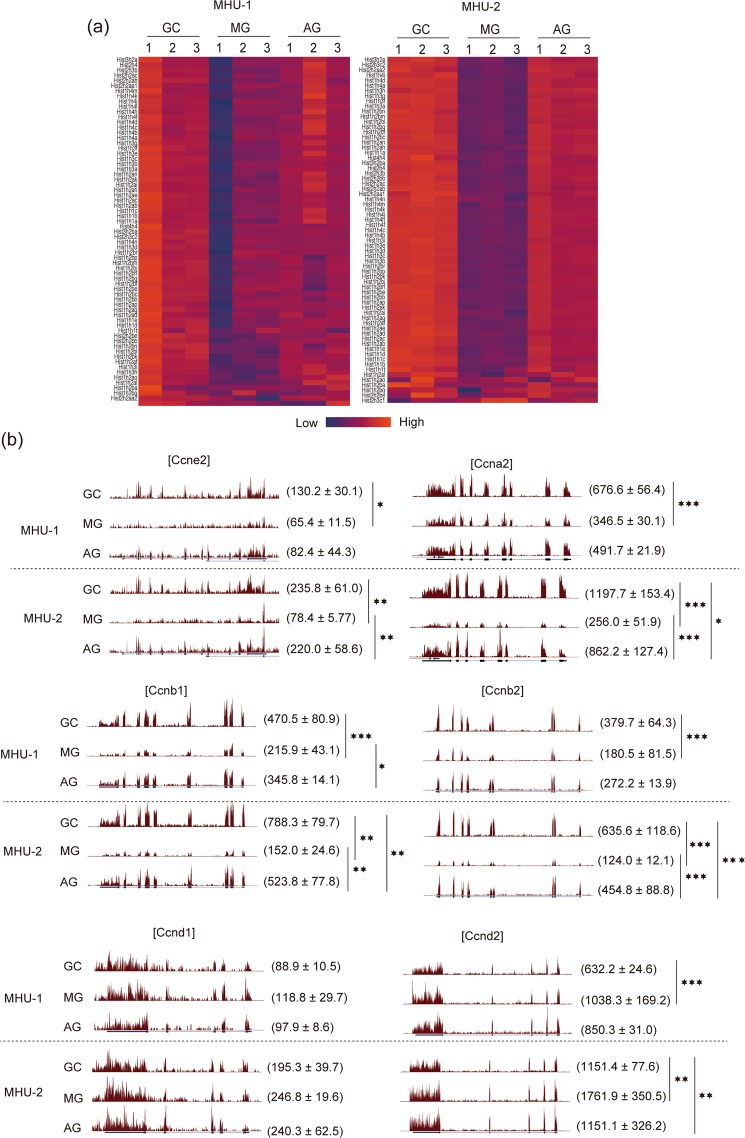
Expression of cell cycle-related genes in the thymus of mice from spaceflight and ground control experiments. (**a**) Heat map of histone gene expression in the thymus of GC, MG and AG mice. Up- and down-regulated genes are indicated as red and blue, respectively. (**b**) RNA-seq tracks of typical cyclin genes (*Ccne2, Ccna2, Ccnb1, Ccnb2, Ccnd1, and Ccnd2*). Upper panels are tracks of MHU-1 and lower panels are that of MHU-2. Parentheses show average values of normalised gene expression with standard deviation. ***P < 0.001, **P < 0.01, and *P < 0.05 of FDR.

Down-regulated genes in the MG group included many histone genes (Fig. 4a). As expression of histone genes are known to be positively correlated with cell proliferation^31^, this implies that spaceflight could cause a reduction of cell proliferative activity. Consistent with this, in addition to histone genes, expression of cyclins controlling progression of G2, M and S phase (Ccna2, Ccnb, and Ccnb2) were down-regulated by spaceflight (Fig. 4b). Moreover, the reduction of cell cycle-regulating genes was partially rescued by artificial 1 × *g* exposure during spaceflight (Fig. 4b). Overall, RNA-seq analysis suggests that spaceflight leads to a reduction in mitotic cells in the thymus.

### Expression of tissue-specific genes was increased in the thymus of mice exposed to spaceflight

**Figure 5:**
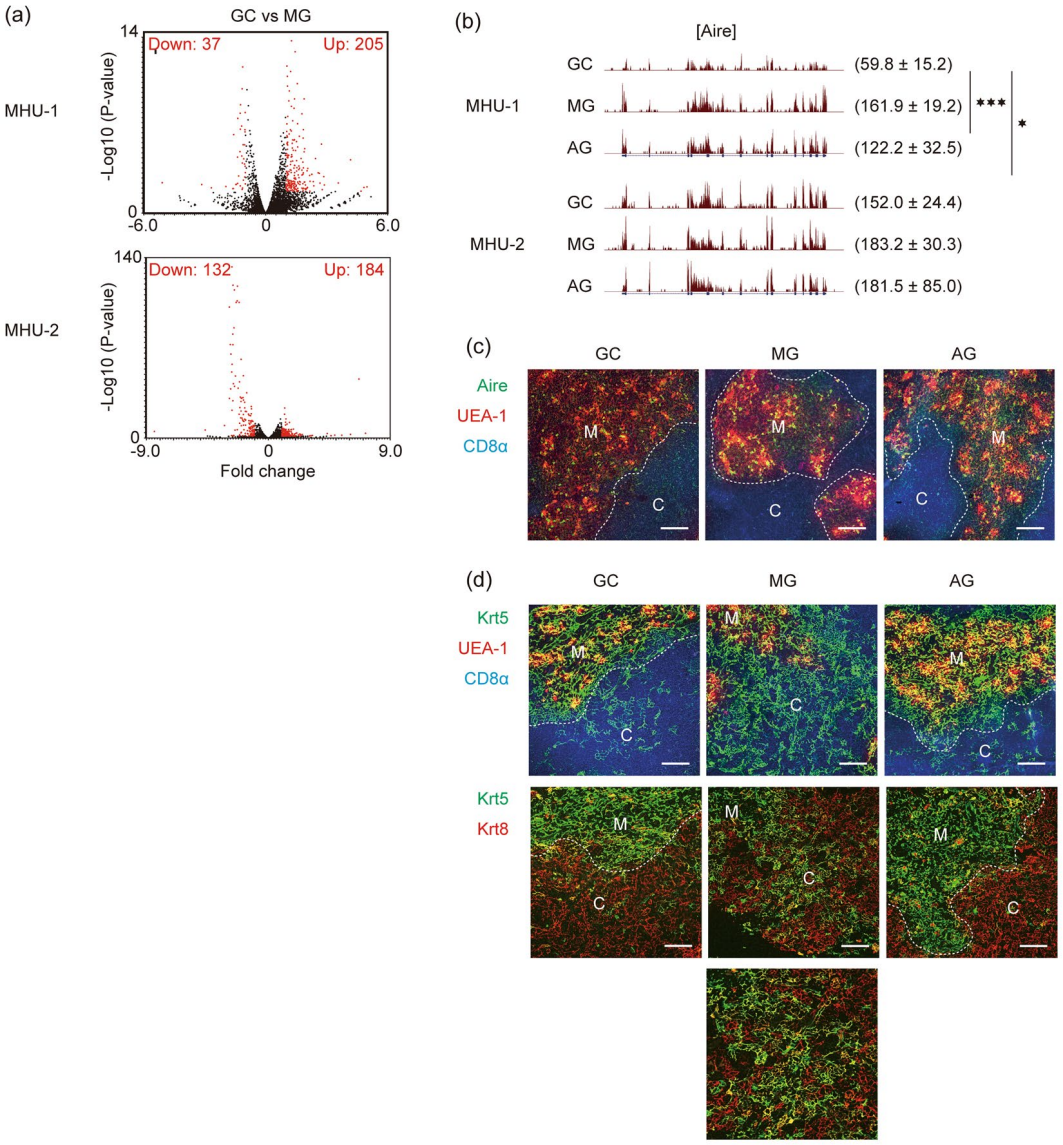
Analysis of genes up-regulated in the thymus of mice from spaceflight and ground control experiments. (**a**) Volcano plots of RNA-seq data of TSG in MHU-1 and MHU-2. Significantly up- or down-regulated TSGs (P < 0.05, two-fold change) are indicated as red dots. (**b**) RNA-seq tracks of *Aire* gene. Upper track is MHU-1 data and lower track is MHU-2. Parentheses show average values of normalised gene expression with standard deviation. ***P < 0.001, and *P < 0.05 of FDR. (**c**) Immunohistochemical staining of thymic sections from GC, MG and AG mice with anti-Aire (green), UEA-1 -lectin (red) and anti-CD8α (blue). UEA-1 is a marker of mTECs, scalebars 100 µm. M and C in panels indicate medulla and cortex, respectively. Dotted lines indicate the border of medulla and cortex. (**d**) Immunohistochemical staining of thymic sections from GC, MG and AG mice with a combination of anti-Krt5 (green), UEA-1 (red) and CD8α (blue) (upper panels) and a combination of anti-Krt5 (green) and anti-Krt8 (red) (middle and lower panels). Part of MG panel is enlarged in a lower panel. Scalebars, 100 µm. M and C in panels indicate medulla and cortex, respectively. Dotted lines indicate the border of medulla and cortex. The medulla and cortex border of MG samples was difficult to define from these sections.

Analysis of up-regulated genes suggested that various genes expressed in a tissue specific manner (TSG) were commonly up-regulated in the thymi of MG mice during MHU-1 and MHU-2 compared with respective GC counterparts (Fig. 5a). Because medullary thymic epithelial cells (mTECs) are known to ectopically express many TSGs, it is likely that the increased TSGs were derived from mTECs. Statistical analysis (binominal distribution) of the data confirmed that TSGs were enriched in the up-regulated gene set (P = 4.07 × 10^−19^ in MHU-1, P = 1.68 × 10^−12^ in MHU-2), but not in the down-regulated gene set (P = 0.748 in MHU-1 and P = 0.675 in MHU-2) (Fig. 5a). Ectopic TSG expression in mTECs is regulated by the transcription factor autoimmune regulator (Aire)^32^. However, expression of Aire was not reproducibly changed in the two missions (Fig. 5b). Moreover, immunostaining showed no considerable increase in Aire-expressing mTECs following spaceflight (Fig. 5c), suggesting that other mechanisms could be involved in this up-regulation of TSG expression.

Because TSG expression was most likely increased by an Aire-independent mechanism, we investigated the thymic epithelial cells in the spaceflight samples in more detail. Careful evaluation of thymic sections suggested that some K5-positive medullary thymic epithelial cells were mislocalised in the cortex region the thymi from MG mice (Fig. 5d). Thus, exposure to microgravity might affect homeostatic mechanisms maintaining thymic epithelial cells, which may lead to a change in gene expression.

## Discussion

The thymus is sensitively affected by various stressors including gravity changes^15–17,33,34^. Indeed, spaceflight causes a reduction in newly generated T cells in the blood of astronauts^24^, most likely due to the impairment of thymic function due to atrophy. Consistently, ours and other studies indicated thymic atrophy in mice after spaceflight. Our data further suggested that a reduction in thymic cell proliferation might be the leading cause of spaceflight-induced thymic atrophy. Thymic atrophy appears to be classifiable into two types^17^. The first type is caused by apoptosis of thymic cells, which is frequently induced by up-regulated serum glucocorticoid. The second type is ascribed to the reduction of TECs, which is caused by aging, pregnancy, and infections. Interestingly, immunostaining showed that localisation of mTECs was affected after spaceflight. Thus, it is possible that a relatively long-term spaceflight could disturb some TEC function, thereby leading to a reduction in thymic cell proliferation. To clarify the mechanism, it would be necessary to analyse gene expression and other parameters of isolated TECs and thymocytes from the thymus after spaceflight in the future.

The relative thymus size in AG mice was significantly smaller than that in GC mice. Thus, besides gravity, other factors might influence the thymic size during spaceflight missions. AG mice experienced many environmental changes during the mission (i.e. space radiation, hypergravity and stress at launching and landing, high CO_2_ concentration, transportation to the tissue collection site after landing) as compared with GC mice. These are summarised in Supplementary Table 1. Thus, other causes of reduction in thymic size during space missions need to be determined in the future to fully understand the impact of space travel on the thymus.

Spaceflight and altered gravity changed the gene expression profile in the thymus^35,36^. Previous comprehensive microarray gene expression analysis suggested that spaceflight changes expression of genes regulating stress, metabolism of the glucocorticoid receptor, and T cell signalling activity in the murine thymus^35^. Although our RNA-seq data showed a change in expression of many genes in the thymus, the list of affected genes was considerably different from the previous gene list. Moreover, gene ontology analysis of the differential genes did not suggest terms related to the previous conclusion. This apparent discrepancy might be due to differences in flight schedules as summarised in Supplementary Table 1. In the previous study, mice were flown in the space shuttle for 13 days. In contrast, we analysed mice staying for 35 days and 30 days on board the ISS. This relatively long-term flight on the ISS may lead to further changes in gene expression in the thymus compared with the early impact caused by the space environment.

Previous PCR analysis of 84 cancer related genes revealed a change in expression of 15 genes (11 up-regulated genes and four down-regulated genes) in the thymus of mice flown in space for 13 days^22^. Pathway analysis of the gene set implied that spaceflight might influence apoptosis and cell cycle checkpoint pathways. Consistently, it was also shown that DNA fragmentation detected by TUNEL assay was enhanced in the thymus of the mice experiencing spaceflight. Our comprehensive RNA-seq analysis also suggests an impact on cell cycle progression in thymic cells. However, we did not find significant signs of apoptosis enhancement in the altered gene list. Considering the flight schedule, one possible explanation is that apoptosis of thymic cells transiently occurs during the early phase of a spaceflight, and subsequent inhibition of thymic cell proliferation starts and persists in the later stages.

Foetal thymic organ culture (FTOC) is an *in vitro* experimental procedure to investigate the development of T cells. In a previous study, FTOC was used to evaluate the effect of vector-averaged gravity on T cell development in a clinostat^37^. In that study, flow cytometric analysis showed that T cell development was impaired by decreasing CD4^−^CD8^−^ (double negative; DN) cells^37^, which are precursors of mature T cells in the thymus. Although a complete defect in mature T cell development generally causes a severe reduction of the medulla area in the thymus^38^, immunostaining suggested that the area of the thymic medulla was not affected by spaceflight. Notably, given that a portion of the DN cell population is highly proliferative^39^, it is possible that spaceflight and gravity change might partially impair the proliferation of DN cells. Thus, a partial defect in the proliferation of DN cells could cause a reduction in the total thymocyte number, thereby leading to a reduction in thymic size.

Because spaceflight is extremely expensive, reproducibility is one of the concerns in the spaceflight experiments. In this study, we performed RNA-seq analysis of the thymus from two independent flight experiments, MHU-1 and MHU-2. Importantly, we used a common platform of cDNA preparation, deep-sequencing, and data analysis for these two samples to minimise the technical deviation. Indeed, PCA analysis showed that GC samples were similar between MHU-1 and MHU-2. Notably, in contrast to GC samples, gene expression profiles of flight samples (AG and MG) were considerably different between the two missions. This might be ascribed to the differences in flight schedules and conditions (Supplementary Table 1). The factor critically affecting gene expression in the thymus remains unclear, but our data indicate that the differences in flight conditions and schedules should be considered in the interpretation of space experiments.

MARS provides a unique platform to investigate the effect of artificial gravity during spaceflight^26,27^. Previous studies showed that exposure to 1 × *g* alleviated bone loss and retinal apoptosis of vascular endothelial cells caused by spaceflight^26,40,41^. Our data suggested that thymic atrophy after spaceflight was significantly mitigated by 1 × *g* exposure during spaceflight. Several countermeasures (i.e. nutritional supplementation, exercise, pharmacological interventions) against immune dysregulation during spaceflight have been proposed^42,43^. Given that spaceflight impairs the thymic function in humans^24^, artificial 1 × *g* could be used as a countermeasure for alleviating impaired thymic function during spaceflight. Notably, because of a gravity gradient caused by the relatively small radius of cage rotation (15 cm), mice might still experience lower gravity on the upper side of the body, especially when they stood perpendicular to the gravity gradient, which may explain the difference between AG and GC animals. However, interestingly, this may also suggest that the countermeasure of whole body exposure to artificial 1 × *g* might not be necessary, but a partial artificial gravity could be sufficient. Future works using MARS will clarify the benefits of artificial gravity and its threshold in other organ functions affected by spaceflight.

In conclusion, this study showed that impaired proliferation of thymic cells might be a cause of the thymic atrophy observed in relatively long-term spaceflight. Moreover, this impact on the thymus could be significantly alleviated by exposure to artificial 1 × *g* during spaceflight. This result might be useful for health management during and after long-term spaceflights in the future.

## Materials and Methods

### Mice

All mouse experiments were approved by the Institutional Animal Care and Use Committee of the University of Tsukuba, JAXA, Explore Biolabs, and NASA, and were conducted according to the applicable guidelines in Japan and the United States of America. Mice were maintained under specific pathogen-free conditions. The mice and treatment under space and ground experiments were described previously^14,26,27^. Briefly, pre-acclimation of mice was conducted in the Space Station Processing Facility Science Annex at Kennedy Space Center (KSC) in Florida, USA. Twelve C57BL/6 J male mice (8-week-old for MHU-1 and 9-week-old for MHU-2) in transportation cage units (TCU) were launched aboard the SpaceX rocket from the KSC and transported to the ISS. On board, mice were transferred to habitat cage units (HCU) and housed singly under AG (1 × *g* on the bottom floor of the HCU, at a centrifugation speed of 77 rpm) or MG environments. In the return process, all mice were transferred to TCUs, and the Dragon vehicle loaded with the TCUs was splashed down in the Pacific Ocean off the coast of California, USA. Returned TCUs were transported to a port in Long Beach by ship, and then to the tissue collection site by road. Housing and transport conditions for MHU-1 and MHU-2 are summarised in Supplementary Table 1. The ground control experiment replicating the housing conditions of the flight experiment was conducted at the JAXA Tsukuba Space Center in Japan. Following euthanasia, thymi were removed and cut, and a quarter of the thymi was snap-frozen in OCT compound (Sakura Finetek, Japan) and a second quarter of the thymus was used for RNA preparation. Thymi for RNA preparation was quickly frozen in liquid N_2_, and kept in dry ice and then in a −80 °C freezer.

### Immunostaining of tissue sections

Sections (6 µm thickness) of the thymus in OCT compound were mounted on glass slides coated with amino silane and fixed with ice cold acetone for 5 min. The sections were blocked with 10% anti-goat serum in phosphate buffered saline (PBS) and then treated with primary antibodies in PBS containing 10% goat serum for 1 h at room temperature or overnight at 4 °C. After washing and further incubating with fluorescence-labelled secondary antibodies for 1 h, the sections were covered with glass coverslips using mounting solution. Images were obtained using Keyence and a Leica confocal laser scanning microscope. Three different sections were analysed for each sample.

### Enzyme-Linked Immunosorbent Assay (ELISA)

Concentration of corticosterone in murine plasma was measured by using corticosterone EIA Kit (Cayman Chemical, Ann Arbor, MI) according to manufacturer’s protocol.

### RNA-Seq analysis

The RNA-Seq method was performed as previously described. Briefly, total RNA was extracted from the thymus using TRIzol reagent according to the manufacturer’s protocol (Thermo Fisher Scientific, Waltham, MA). The RNA-Seq library was prepared by using the NEBNext Ultra Directional RNA Library Prep Kit (New England Biolabs [NEB], Ipswich, MA) after depleting rRNA (NEBNext rRNA Depletion Kit; NEB). Paired-end sequencing (2 × 36 bases) was performed by using NextSeq500 (Illumina, San Diego, CA). FASTQ files were processed using Fastp^44^. Sequence reads were mapped using CLC Genomics Workbench (Version 11.0.1; Qiagen, Redwood City, CA). Differential expression was analysed by empirical analysis using the Empirical Analysis of DGE tool (edgeR test) in CLC Genomics Workbench and CLC Main Workbench.

### Statistics

Student’s t-test was used for determining P-values. The Exact Test of Robinson and Smyth was used for RNA-Seq data analysis, and the FDR-corrected P-value was used for testing statistics. TSG list reported previously^45^ was used for the analysis.

## Supplementary information

Supplementary Table 1

## Data Availability

All data that support the findings of this study are available from the corresponding author upon reasonable request. RNA-Seq data are deposited in DDBJ (DRA008961).
